# In what ways do emerging adults with substance use problems experience their communities as influencing their personal recovery processes?

**DOI:** 10.1002/jcop.22816

**Published:** 2022-02-20

**Authors:** Nina Kavita Heggen Bahl, Emil Øversveen, Morten Brodahl, Hilde E. Nafstad, Rolv M. Blakar, Ottar Ness, Anne S. Landheim, Kristin Tømmervik

**Affiliations:** ^1^ Department of Research and Development, Clinic of Substance Use and Addiction Medicine St. Olavs University Hospital Trondheim Norway; ^2^ Department of Sociology and Political Science Norwegian University of Science and Technology Trondheim Norway; ^3^ Mental Health Division, Norwegian National Advisory Unit on Concurrent Substance Abuse and Mental Health Disorders Innlandet Hospital Trust Brumunddal Norway; ^4^ Department of Psychology University of Oslo Oslo Norway; ^5^ Department of Education and Lifelong Learning Norwegian University of Science and Technology Trondheim Norway; ^6^ Mental Health Division, Norwegian National Advisory Unit on Concurrent Substance Abuse and Mental Health Disorders Innlandet Hospital Trust Brumunddal Norway; ^7^ Campus Elverum Innlandet University of Applied Sciences Elverum Norway; ^8^ Department of Research and Development, Clinic of Substance Use and Addiction Medicine St. Olavs University Hospital Trondheim Norway

**Keywords:** collaborative research, emerging adulthood, psychological sense of community, recovery, substance use problems

## Abstract

Applying the multiple psychological sense of community concept (MPSOC), this study explored how emerging adults with substance use problems experience the influences of various senses of community and communities on their personal recovery processes. Semi‐structured interviews with 21 emerging adults from different urban contexts in Norway were analysed using a collaborative, seven‐step, deductive, and reflexive thematic approach. MPSOC is shown to be a key concept for achieving a broad, in‐depth understanding of emerging adults' senses of community and personal experiences of community influences on recovery processes from substance use. Positive and negative senses of community in geographical, relational, substance use‐related and ideal communities influence the potentials and challenges in emerging adults' recovery processes. Supportive and motivating community relationships, meaningful activities with peers, and distance from recovery‐impeding communities were identified as important recovery components. To promote recovery and prevent substance use in emerging adults, community approaches and tools applied in substance use treatment have to take into account and utilise multidimensional and age group‐specific aspects of belonging.

## 1 INTRODUCTION

Social relationships and social integration fulfil some of our most fundamental needs, like our need to be part of, and to feel a sense of belonging (Baumeister & Leary, 1995; Brewer, 2004; Fiske, 2018; Sarason, 1974). We all depend on social relationships to ensure social integration, and thereby health and well‐being, through our lifespans (Bahl et al., 2021). A good life is about belonging to and being a contributing member of groups, communities, and society (Bahl et al., 2017; Nowell & Boyd, 2010). It is essential to recognise our need to belong, be connected to, and be part of social relationships and social contexts (Brewer, 2004). Across our lifespans, humans need to be accepted by, connected with, and be of value to others (Bahl et al., 2021b; Cicognani et al., 2014; Moore et al., 2018; Myers, 2004; Sarason, 1974). For treatment of problematic substance use, it is highly relevant to know more about individuals' social contexts and types of social bonding, as social relationships and social bonding can ultimately contribute to prevent and reduce misuse behaviours and promote recovery (Bathish et al., 2017; Ferrari et al., 2002; Kelly et al., 2014; Lardier Jr et al., 2017; Mayberry et al., 2009; Moore et al., 2018; Mudry et al., 2019; Stevens et al., 2010, 2012; Wenaas et al., 2021).

Emerging adulthood is a particularly vulnerable period of life, with changing social structures and relationships (e.g., family, residential community, school, and peers) (Arnett, 2000; Bahl et al., 2021b). Thus, during this life phase, central elements needed for recovery may be missing; namely, social integration and belonging to groups, communities, and society at large (Bathish et al., 2017; Jason et al., 2001; Kelly et al., 2014; Moore et al., 2018; Mudry et al., 2019; Stevens et al., 2012; Wenaas et al., 2021). Furthermore, in many countries this developmental period is also marked by the highest prevalence of risk‐related episodes of high alcohol consumption and substance use (Arnett, 2000; Baumer et al., 2018; Bergman et al., 2016; Norwegian Institute of Public Health, 2018). We currently lack sufficient knowledge to prevent the social marginalisation of, or promote recovery among, this at‐risk group (Foster & Spencer, 2013). To address this issue, it is essential to understand emerging adults' sense of community and how their communities influence their personal recovery processes. Using the concepts of psychological sense of community (PSOC) (McMillan & Chavis, 1986; Sarason, 1974) and multiple psychological senses of community (MPSOC) (Brodsky et al., 2002; Mannarini et al., 2014), the purpose of this study is to provide knowledge about groups, community, belonging and recovery among emerging adults with substance use problems at particular risk of social marginalisation – those who are neither in education nor in employment. We do so by analysing data from a larger, national study conducted on assignment from the Norwegian Directorate of Health regarding service experiences among emerging adults with substance use problems aged 18–23 years, a subgroup defined by the Norwegian Directorate of Health as a ‘special attention and priority group' for Norwegian mental health and addiction services.

### 1.1 Study aims and research question

Our research question was: In what ways do emerging adults with substance use problems experience their communities as influencing their personal recovery processes? We approached the current lack of age group‐specific research within both the PSOC and substance use recovery fields (Foster & Spencer, 2013; Hennessy, 2017; Townley et al., 2011) by studying emerging adults' PSOC and recovery processes. We also addressed the mismatch between the facts that (a) those with substance use problems are members of multiple communities that can simultaneously influence their recovery (Bahl et al., 2019, 2021a; Mayberry et al., 2009; Moore et al., 2018) and (b) the restricted focus in research on community influence on substance use recovery on only one single community reference, and then typically sober living contexts and therapeutic communities. Herein, we studied the influences of different community types and senses of community in processes of personal recovery from substance use problems. To our knowledge, our previous study of MPSOC and recovery among those receiving services for problematic substance use (Bahl et al., 2019) is the only such study to date. It is also the only PSOC and substance use recovery study to have included the voices of those with substance use problems. The findings from that study indicated that all dimensions of the broader MPSOC concept—experiences of both positive and negative connections within geographical and relational communities—take part in recovery processes among those receiving services for problematic substance use (Bahl et al., 2019). Moreover, ideal communities were identified as central to individuals' descriptions of community relationships and recovery. To better understand the role different communities play in substance use recovery during a specific, vulnerable life phase, the current follow‐up study of emerging adults was needed.

To sum up, we aimed to provide an in‐depth understanding of how multiple communities (e.g., family, friends, service‐related, geographical, ideal recovery communities) are experienced as shaping and influencing the personal recovery processes among emerging adults who receive services for substance use problems from urban municipalities in the Scandinavian welfare state, Norway.

## 2 EMERGING ADULTS' PSYCHOLOGICAL SENSE OF COMMUNITY

PSOC refers to our meaning systems of being part of, connected to and supported by a community; it also encompasses the values of caring, compassionate relationships, communities, and social responsibility (Bahl et al., 2021b; Brodsky, 2009; Kloos et al., 2012; McMillan, 1996; Nowell & Boyd, 2010; Sarason, 1974). Today's most widely applied conceptualisation of PSOC is by McMillan and Chavis, whose point of departure is that positive PSOC consists of four conceptual dimensions (Chavis et al., 2008; McMillan & Chavis, 1986): (a) a feeling of belonging and identification with the community (membership); (b) integration and fulfilment of one's needs through the community's or members' resources, while simultaneously making one's own contributions to the community (fulfilment of needs); (c) a sense or feeling of having some influence on the community and experiencing an acceptable influence from the community (mutual influence); and (d) a sense that members of the community share, and will continue to share, a common history (shared emotional connection). In addition to these core dimensions, PSOC has been extended by the MPSOC concept, with respect to multiple community references (geographical, relational and ideal communities) and affective states (i.e., positive and negative PSOC) (Bahl et al., 2019; Brodsky et al., 2002; Mannarini et al., 2014).

Negative psychological sense of community (NPSOC) is a centrifugal force that symbolically moves individuals away from the community (Brodsky, 1996), and has been operationalised by four dimensions: (a) a need to distinguish oneself from the community and its members, an experience of being different from other community members, and a refusal to be associated with anyone who belongs to the community (distinctiveness); (b) a passive, uncaring attitude toward the community and its shared events, and a trend to refrain from any activities with other community members (abstention); (c) a feeling that the community and its members are a source of frustration (frustration); and (d) a feeling of being extraneous, unfamiliar and unrelated to the community, its members and its shared traditions or history (alienage) (Mannarini et al., 2014). The PSOC concept's various dimensions focus on our social origins, recognising that we must, in some way, be connected to and value other people and social groups (Brewer, 2004). Few studies to date have focused on the negative affective dimension. These studies suggest that an NPSOC can be both destructive and socially isolating, as well as socially adaptive and leading to positive outcomes for individuals who perceive their community as a burden rather than a resource (Brodsky, 1996; Lardier Jr et al., 2020).

### 2.1 PSOC and age

Over the past two decades, several studies have considered the importance of communities and PSOC for health and well‐being within specific age groups. These have focused primarily on adolescents and older adults (Bahl et al., 2021b; Chiessi et al., 2010; Li et al., 2014; Prati et al., 2021). The few PSOC studies of young people that have included those with substance use problems have primarily assessed students in the United States (Gordon et al., 2020; Henry & Slater, 2007; Mayberry et al., 2009) or young minority groups (e.g., Hispanic students and students of colour) (Lardier Jr et al., 2017, 2019a; 2019b). Results from these studies suggest that positive geographical (i.e., neighbourhood) PSOC can have a key moderating effect on predictors of substance use (e.g., social disorganisation, violent, and deviant behaviour, family conflict). They also indicate that supportive adults (e.g., adult mentor relationships, adult allies) and social support systems (e.g., parental, familial, peer, school, and community‐based organisations) are important for young peoples' positive development and well‐being. These factors also reduce the effects of negative developmental experiences (e.g., on mental health, substance use problems) (Gordon et al., 2020; Lardier Jr et al., 2017; Schmidt et al., 2007). Moreover, strong, positive community connections are especially important when care and support at home are limited (Moore et al., 2018). Furthermore, multiple ecological relationships and contexts (e.g., parental support, neighbourhood PSOC, school belongingness) are central to creating belongingness and reducing substance use, and thus initiate positive developmental processes and outcomes (Gordon et al., 2020; Henry & Slater, 2007; Lardier Jr et al., 2017, 2019a, 2019b; Mayberry et al., 2009; Moore et al., 2018).

Emerging adulthood has been proposed by Arnett (2000, 2007) to be a distinct developmental stage, being neither adolescence nor young adulthood. This period spans the late teens through twenties (first introduced as 18–25 years, it is now often extended to 29 years). Emerging adults are a particularly vulnerable group with respect to community belonging and substance use (Arnett, 2000; Bahl et al., 2021b; Bergman et al., 2016). Individuals in this life phase can be more occupied with themselves and their own situation; their community involvement tends to be relatively low (Arnett, 2012; Weiss‐Dagan, 2021). Thus, emerging adults can feel less socially integrated and have a lower sense of belonging to communities and society. With respect to substance use, this period marks the highest prevalence of risk‐related episodes of high alcohol consumption and substance use (Arnett, 2000; Baumer et al., 2018; Bergman et al., 2016; Norwegian Institute of Public Health, 2018). Yet few studies have addressed central elements needed for recovery in this developmental stage: different senses of community in various communities and social environments. Moreover, we know little about emerging adults with substance use problems who are not part of typical communities, such as school or work, and who are at risk of becoming socially marginalised adults. Thus, MPSOC research with this population provides a central opportunity for community psychology globally to gain the knowledge to fulfil its core agenda of health‐related prevention and promotion. Such research may prevent social marginalisation and substance use problems, and promote recovery and meaningful lives without problematic substance use, among those approaching adulthood.

## 3 THE INFLUENCE OF COMMUNITIES ON RECOVERY FROM SUBSTANCE USE DURING EMERGING ADULTHOOD

Studies of processes of recovery from problematic substance use have shown the importance of citizenship, supportive relationships, and experiencing social bonding and a sense of community (Bathish et al., 2017; Jason et al., 2001; Kelly et al., 2014; Moore et al., 2018; Mudry et al., 2019; Stevens et al., 2012; Wenaas et al., 2021). These studies have generally defined recovery as both a personal process and a social transformation. Simultaneously, recovery processes are initially formed and take place within people's everyday lives. Both meaningful activities and multiple supportive relationships are crucial factors in recovery processes (Brekke et al., 2020; De Ruysscher et al., 2017; Ness et al., 2014; Panel, 2007). However, different communities can also present unique challenges to processes of recovery from substance use problems, for example, exposure to substance use, violence, and trauma (Bahl, 2019; Gonzales et al., 2012; Lardier Jr et al., 2017; Weston et al., 2018). To date, little is known about how different communities influence processes of recovery from substance use problems during emerging adulthood (Goodman et al., 2011; Mawson et al., 2015). Among the major criteria for the transition to adulthood are accepting responsibility for oneself and making independent decisions (Arnett, 2000). Thus, in their transition to adulthood, it is important for emerging adults to depend on support and help from others, and their communities, to take responsibility, and make adequate decisions about the personal demands of a recovery process.

Taking as our point of view that emerging adults are members of multiple ecological relationships and social contexts, where different communities can represent sources of positive and negative PSOC, as well as recovery potentials and challenges, this study aimed for a complete and in‐depth understanding of what ways emerging adults with substance use problems experience their communities as influencing their personal recovery processes.

## 4 MATERIALS AND METHODS

This study was a qualitative follow‐up study of a previous, in‐depth collaborative MPSOC and recovery study among those with substance use problems who received services from Norwegian municipalities (Bahl et al., 2019). Like the previous study, this one used a collaborative research design, as well as reflexive thematic deductive analyses based on MPSOC as an integrative theoretical framework. This thematic analysis included several relevant perspectives: community psychological (first author), sociological (second author), and peer researcher perspective (third author).

### 4.1 Collaborative research design

Within substance use research, a growing aspiration is to involve those with personal experience from substance use problems and recovery throughout the research process (Faulkner, 2004; Nowotny et al., 2001; Trivedi & Wykes, 2002). In this study, the perspectives of individuals who were experiencing, or had experienced, recovery from substance use problems were included in collaboration at several stages of the research process.

First, a peer support worker from the Drug and Alcohol Competence Centre in Central Norway participated on the project planning board, in planning the data collection and developing the initial interview guide. This guide was later adapted for emerging adults by the first author and collaborators at the Clinic of Substance Use and Addiction Medicine. Second, the sample of 21 participants had experiences with both pre‐ and in‐recovery processes: Nine participants still used substances with which they had problems and 12 had stopped using substances at the time of study participation (see Table 1). Third, a peer researcher (third author) collaborated with the first and second authors on the analysis. This peer researcher also had recovery experiences, having personal experience of recovering from substance use problems, worked with emerging adults who were recovering from substance use problems and having been educated in and experienced qualitative analysis methods within the field of substance use and addiction.

**Table 1:** Participants' backgrounds and community belongings

Participant	Interviewer	Age	Region	Healthcare services for substance use problems	Current use of substances?	Problematic substances	Relational communities	Geographical communities	Ideal communities	Substance use‐related communities
M20 (N1)	1	20	North	Financial assistance from NAV; Drug and addiction Services; Rehabilitation centre	No	Cannabis, cocaine, alcohol	Nuclear family + Friends +		Meaningful activities	Nuclear family, friends and acquaintances +
M21 (N21)	1	21	North	Municipal housing; Financial assistance from NAV; Unit for mental health and addiction; Psychologist, Interdisciplinary group, Low‐threshold offer for people with substance use problems	Yes	Alcohol, amphetamine	Nuclear family + Low‐threshold offer for people with substance use problems			
M22 (N3)	7	22	North	Unemployment benefits from the Norwegian Labour and Welfare Administration; Outreach service for emerging adults (18–23); Low‐threshold offer for people with substance use problems	No	Cannabis	Virtual (gaming) + Nuclear family +/‐ Low‐threshold offer for people with substance use problems +			Friends and acquaintances ‐
W19 (W1)	1	19	West	Financial assistance from NAV; Outreach service for emerging adults (18–23) (activity group); Work training from the NAV	No	Polysubstance use	Outreach service for emerging adults (18–23) (activity group) + Nuclear family +			Partner + Friends ‐
W22 (W2)	1	22	West	Work assessment allowance from NAV; Specialist healthcare service; housing support; Outreach service for emerging adults (18–23) (activity group); Financial support, Music therapy	Yes	Cannabis, amphetamine	Nuclear family+/‐Activity group +		Music therapy	Nuclear family and acquaintances ‐/+
M23 (W3)	1	23	West	Work assessment allowance from NAV; Municipal housing, Specialist healthcare service; Centre for mapping and follow‐up; Housing support	No	Polysubstance use	Nuclear family ‐Friends+/‐	Municipal housing ‐	Music therapy	
W20 (W4)	1	20	West	Centre for victims of sexual abuse; PUT; Financial assistance from NAV; Outreach service for emerging adults (18–23) (activity group)	Yes	Cannabis	Nuclear family +/‐ Outreach service for emerging adults (18–23) (activity group) +			Friends ‐
M22 (W5)	1	22	West	Work assessment allowance from NAV; Psychologist (interdisciplinary specialised substance use treatment); Psychologist (mental healthcare); Financial adviser; Interdisciplinary specialised substance use problem treatment; Physical training in outpatient clinic	No	Polysubstance use	Nuclear family +	Treatment institution (interdisciplinary specialised treatment of substance use problems) ‐	Meaningful activities	Acquaintances ‐
M23 (W6)	1	23	West	Work assessment allowance from NAV; Interdisciplinary specialised substance use problem treatment; Psychologist (mental healthcare); Financial support; Physical training in outpatient clinic	No	Cannabis, amphetamine	Peers at interdisciplinary specialised substance use problem treatment +	Municipal housing ‐ Treatment institution (interdisciplinary specialised substance use problem treatment) +/‐		Friends +
W20 (W7)	2	20	West	Financial assistance from NAV; Outreach service for emerging adults (18–23) (activity group); Psychologist	No	Polysubstance use	New friends + Partner + Nuclear family +		Creative activities and animal therapy	Friends ‐/+
W20 (W8)	2	20	West	Financial assistance from NAV; Outreach service for emerging adults (18–23) (activity group and work training); Psychologist	No	Polysubstance use	Nuclear family + Outreach service for emerging adults (18–23) (activity group and community at work) + Partner +			
M18 (E3)	4	18	East	Child welfare services; Addiction consultant	Yes	Cannabis	Nuclear family +/‐		Work	Friends +
M23 (E23)	3	23	East	Outreach service for emerging adults (18–23)	Yes	Cannabis	Nuclear family +/‐Partner +			Friends ‐
M22 (E3)	4	22	East	Outreach service for emerging adults (18–23); Work training/preparation from NAV, Housing	No	Cannabis	Nuclear family +/‐ Friends +		Meaningful activity	
M19 (E4)	5	19	East	Outreach service for emerging adults (18–23)	Yes	Cannabis	Outreach service for emerging adults (18–23) +			
M21 (E5)	4	21	East	Financial assistance from the NAV; Outreach service for emerging adults (18–23)	No	Cannabis	Nuclear family +		Youth leisure clubs/activities and meeting places for youth Music therapy	Friends ‐
M23 (E6)	6	2	East	Child welfare services (case worker/housing), Outreach service for emerging adults (18–23); Psychologist (mental health)	No	Cannabis, alcohol	New friends + Nuclear family ‐ Ethnic community ‐		Dance training	Friends ‐/+
M22 (E7)	4	22	East	Financial assistance from the NAV; Outreach service for emerging adults (18–23)	Yes	Cannabis, cocaine	Friends + Nuclear family +			
M20 (E8)	4	20	East	Outreach service for emerging adults (18–23)	Yes	Cannabis	Outreach service for emerging adults (18–23) (activities) + Friends Partner		Meaningful activities (work, football, physical exercise)	Work (selling substances)
M23 (E9)	4	23	East	Outreach service for emerging adults (18–23); Financial assistance; Temporary housing and work training/preparation from the NAV	Yes	Cannabis	Friends +		Meaningful activity (work or courses) and youth leisure centres	
W20 (E10)	3	20	East	Child welfare services (aftercare and financial assistance)	No	Cannabis	Friends + Nuclear family			

*Note*: Informants are represented with codes indicating their gender (F for female and M for male), age, and residential region (N = North, W = West, E = East). + is used when the participant spoke about the community in any way related to the four PSOC dimensions (membership, influence, integration and fulfilment of needs or emotional connection). ‐ is used for NPSOC‐related descriptions (distinctiveness, abstention, frustration or alienage). Both signs are used for communities addressed in both ways. No sign indicates that the participant only reported belonging to the community. Communities are those of which the participant was a part at the interview.

Abbreviations: NAV, Norwegian Labour and Welfare Administration. PUT, Specialised interdisciplinary mental health and substance use services for youth.

### 4.2 Approach to enquiry

The authors took care to meet the American Psychological Association standards for qualitative research (Levitt et al., 2017; Levitt et al., 2018). With respect to Braun and Clarke's dimensions for reflexive thematic analysis (Braun & Clarke, 2019; Byrne, 2021; Clarke et al., 2015), this study is deductive in its theoretical approach, as well as epistemologically essentialist and experiential and constructivist in its perspective. In other words, the study's conceptual framework was applied deductively in coding the material. Moreover, a central essentialist assumption was that participants' descriptions are reflections of their articulated experiences. Furthermore, the study's orientation is experiential in its aim to prioritise emerging adults' own accounts of their life experiences. Finally, the study is constructivist in its orientation, as it moved beyond describing the participants' social world, by examining how multiple communities construct the recovery process.

### 4.3 Social context: Services and family support

Most PSOC research among adolescents and young adults has been conducted in the United States. The sociocultural context here was Norway, a Scandinavian welfare state. In the Norwegian public health system, specialist health services are offered at the regional level. Primary health services are organised and delivered by municipalities. As part of the Norwegian clinical substance use treatment pathway individuals are first offered services by the municipality, before being referred to hospital‐based specialised healthcare services, if needed. After completing specialised treatment, the patient returns to municipal services where the recovery process continues. Aside from an initial excess charge of 2460 NOK (approximately 285 USD), all services are offered free of charge. Generally, the family plays a smaller role in the provision of social and economic security and support in Nordic social democracies compared to other Western societies (Esping‐Andersen, 1990). Thus, the participants interviewed may have had different experiences with their communities and social relationships compared with other Western settings and more market‐driven healthcare systems

### 4.4 Material

The study material was interviews, transcribed verbatim, collected as part of a larger national project evaluating service users' experiences with substance use treatment services from the Norwegian municipalities. Conducted by Korus Midt (The Drug and Alcohol Competence Centre in Central Norway) on assignment by the Norwegian Directorate of Health, the larger national project evaluated the effects of a national attempt to upscale and improve health and social services for those with substance use problems (see Korus Midt, 2020a, 2020b, 2020c). The present study utilised data from the national project's second wave, which aimed to generate qualitative knowledge about how emerging adults with substance use problems experience municipal services.

### 4.5 Recruitment and sample

A purposeful sampling strategy was used in three urban contexts to recruit 21 emerging adult participants (inclusion criterion: age 18–23 years) who had received services from the Norwegian municipalities for substance use problems. The Norwegian Directorate of Health has defined the years 18–23 as a particularly difficult, vulnerable transitional life stage during which mental health and addiction services should devote special priority to. All participants were contacted by municipal services staff, including those from primary and specialised healthcare services, the Norwegian Labour and Welfare Administration (NAV) and Utekontakten, a municipal‐level social service aimed at identifying and intervening on behalf of youth at risk of social marginalisation. At their interviews, all participants were receiving municipal services for substance use problems, and represented a variety of community experiences and recovery stages of the recovery process (see Table 1).

### 4.6 Data collection

Study material was collected by seven interviewers across three Norwegian municipalities. For transparency, Table 1 and all excerpts from the material in the Findings and Discussion sections include an interviewer code (1–7). Interviewer 1 is the second author and an academic researcher; Interviewer 2 worked at the Competence Centre for Addiction in the western region; Interviewers 3–6 worked in the Outreach Service for Emerging Adults in the Oslo municipality; and Interviewer 7 worked at the University Hospital of North Norway. All interviewers had either academic training in conducting interviews (6 of 7) and/or clinical competence in communicating with individuals with substance use problems (5 of 7). Individual semi‐structured, in‐depth interviews were conducted. This method was chosen to allow participants to freely describe their experiences with their current life situation, community relationships (e.g., family, friends, and partners), and the municipal services they received. As the data were collected by several interviewers, a semi‐structured guide ensured a consistent overall structure. Participants were specifically asked about their background, current life situation, experiences with municipal services, relationships with family and significant others, and how family and significant others were involved in the services they received.

### 4.7 Data analysis

A seven‐step deductive, in‐depth, reflexive thematic analysis was used in this study. Before this study, the first and second authors conducted a preliminary inductive analysis for a previously published report (see Korus Midt, 2020b), in which it became evident that communities were a key theme. Given the emerging adult participants' descriptions of several communities as important in their recovery processes, it was decided to continue investigating the MPSOC construct's theoretical fit with the emerging adults descriptions, as we had done previously for a general sample of persons with substance use problems (Bahl et al., 2019).

**Figure 1:**
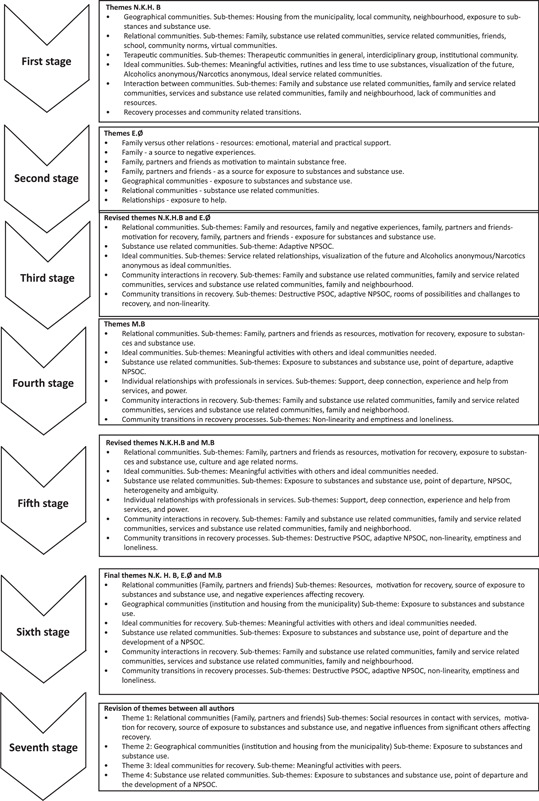
Overview of the development of themes

In steps one and two (see Figure 1), the first and second authors performed individual thematic material analyses from their unique perspectives, using deductive line‐by‐line coding in NVivo 12 (QSR International Pty Ltd.) to capture all positive and negative influences of different communities had on the participants' recovery processes. The third step consisted of meetings during which the two authors shared, discussed, and agreed on the study's main themes. The individual analyses overlapped; The first author's analysis was broader with respect to coding communities and their influences, while the second author's analysis focused specifically on the quality of individual relationships (i.e., between community members) and those relationships' potentials (i.e., possibilities, resources, limitations, and exposure). Overall, this initial collaborative analysis supplied, specified, and concretised the themes, based on both individual analyses. Importantly, it also directed further deductive analysis of the potentials and challenges to participants' recovery processes posed by their communities.

In the fourth and fifth steps, the third author (peer researcher) participated in collaborative analysis meetings. Concretely in the fourth step, the third author read and analysed a selection of the material from his peer researcher perspective. Given the extensive material (452 transcript pages), it was decided that the peer researcher would analyse 10 interviews that contained the most node references from the first author's broad analysis (he also read, but did not specifically analyse, the remaining 11 interviews). This approach to limiting the material analysed has been described as advantageous by peer researchers participating in collaborative research (Pettersen et al., 2019). In the fifth step, the first and third authors met via Zoom Meetings (Zoom Video Communications, Inc.) (due to the COVID‐19 pandemic) to share and discuss themes (the first author shared the themes from step three) until they reached an agreement on the overall themes. This step both nuanced and supplemented the themes from the first collaborative thematic analysis (see Stages 4–5 in Figure 1).

In the sixth step, all three authors collaborating on the analysis met via Zoom Meetings (again, due to the pandemic) to reach an agreement on the final themes (see Stage 6 in Figure 1). Discussions during the fifth and sixth steps were audio‐recorded, allowing the first author to check the arguments toward developing the themes. These six steps subsumed the traditional first five of six steps in a reflexive thematic analysis: familiarisation, coding, searching for themes, reviewing themes, and defining and naming themes.

In the seventh and last step, the final themes were collaboratively reviewed and re‐named by all authors in the writing process of the article.

### 4.8 Ethical considerations

The larger national study was approved by the Data Protection Officer at St. Olavs Hospital in Trondheim, Norway (Reference ID: ESA 17/4211). Consistent with this approval, all participants were informed about what their participation in the study would entail, who would conduct the interview, and that the interview would be digitally audio‐recorded and anonymised before being transcribed verbatim. Finally, before conducting the interviews, each participant was informed that they could withdraw their consent and end the interview at any time. Each participant signed written informed consent before their interview was conducted.

## 5 FINDINGS AND DISCUSSION

### Multiple communities and their influences on recovery processes

As described above, evidence suggests that multiple communities make different contributions and offer different challenges to an individual's process of recovery from substance use problems (Bahl et al., 2019; Dingle et al., 2015; Gordon et al., 2020; Henry & Slater, 2007; Lardier Jr et al., 2017, 2019a, 2019b; Mawson et al., 2015; Mayberry et al., 2009; Moore et al., 2018). Throughout each collaborative reflexive thematic analysis stage, the three researchers identified various community types, which participants described as providing different recovery process potentials and challenges: relational, geographical, ideal, and substance use‐related communities (see Figure 1 for a theme development overview). Detailed below is how participants from a range of urban municipalities described their experiences with multiple communities, as providing different potentials for and challenges to their personal recovery processes. Specifically, we present descriptions of the potentials and challenges participants' communities present in their recovery from substance use. All quotes have been translated by the authors into English, with minor grammatical adjustments for clarity. Informants are represented with codes indicating their gender (F for female and M for male), age, and residential region (N = North, W = West, and E = East).

### 5.1 Theme 1: Relational communities (family, partners, and friends)

#### 5.1.1 Social resources in contact with services

Both parental and peer support have been identified as important for moderating substance use, and in substance use recovery (Gordon et al., 2020; Mayberry et al., 2009; Moore et al., 2018; Ness et al., 2016; Weston et al., 2018). Moreover, autonomy and relatedness are suggested as complementary rather than opposing dimensions in an emerging adult's relationships with their parents (Arnett, 2000; Goodman et al., 2011). During this life stage, it might be presumed that family, partners, and friends are central resources in the substance use recovery process. However, such a presumption is not necessarily accurate in Norway, where young adults are expected to rely more on municipal services than on relational communities as their social support system.

Relational communities were, however, the most prominent theme in the research material. When participants were asked about their everyday social contacts, participants' family, partners, and friends were the relational communities described most often (see Table 1 for more details). Most participants said that these communities played a central role in making their recovery possible. Indeed, family, partners, and friends informed them about, and introduced them to, assisted in contacting and keeping contact with municipal services:

I2: How did you make contact [with services from the municipality]?

F20 (W7): I and my mother called Outreach Service for emerging adults and told them that I needed an appointment, so that I could receive help with economic support of course, and if they could help me with referral to a psychologist or what they could help me with…and then we went down there to talk with them and they suggested that I could join this group and take some steps from there with respect to coming in contact with a psychologist.

I4: Do you have any friends who support you [in your recovery]?

M22 (E3): Yes.

I4: Or do you have a support person for instance?

M22 (E3): No, no support person but friends…yes, who support me and push me forward […] Who say “go to class,” “go to NAV.”

Relational community relationships with family, partners, and friends were also described as providing both practical and emotional support in contact with services:

I4: Do you feel support from having [your mother] with you?

M21 (E5): Yes, yes, my mother is a very strong and good woman…she tells the professionals things as they are and what I need help with and things like that…

I4: Are there any other important persons in your life who have supported you in your process?

M18 (E1): Actually, just my friends… They are the only ones I trust. We support each other.

These quotes suggest that emerging adults, even when primary health services are involved, may still need support to manage, take responsibility for and make decisions about their recovery processes. Among the participants who did not describe support from family, all emphasised support from partners and friends as important to their personal recovery processes. This suggests that a broad understanding of relational communities, including partners and friends, may help to understand recovery facilitators. It also confirms earlier findings that social capital plays an important role in recovery. Finally, it nuances the current understanding, suggesting that social capital may play a central role in emerging adults' recovery from substance use problems, even in an egalitarian social democratic welfare state such as Norway.

#### 5.1.2 Motivation for recovery

Positive social relationships can protect against negative health outcomes. Hope and positive motivation are central elements in this association (Delle Fave et al., 2015; Salsman & Moskowitz, 2015; Stevens et al., 2018). However, there has been little investigation into these relationships. A few previous findings suggest that family and peers are important sources for motivating and initiating recovery from substance use (Wenaas et al., 2021), particularly in emerging adulthood (Goodman et al., 2011). Several participants described family members, partners, and friends as important sources of motivation for recovery, including when family was not directly involved in their recovery process:

I2: Yes, so at that time there was less contact.

F21 (W8): So, that was also the real reason that I asked for help to recover, because I wanted to get back in touch with my family, right…to be included. That was important to me.

I2: Are there any other important persons in your life that you are in contact with?

F20 (W7): There is my new boyfriend who I will live with from next month, when we have been together one year. He is the main reason that I distanced myself from substance use.

I2: Yes, has that been a great motivation?

F20 (W7): He is a very great motivation.

As these quotes illustrate, both family and partner relationships can be central in promoting motivation for recovery. As such, they may be important, not only because they exert pressure to begin the recovery process (see Goodman et al., 2011), but also because these connections and the sense of belonging they offer may contribute to motivation throughout recovery.

#### 5.1.3 Sources of exposure to substances and substance use

Although family, partners, and friends offered central elements for several participants' recovery potential, the same relationships were described by others as introducing challenges, and even impeding their recovery. Among the many participants addressing impeding community relationships, a predominance addressed family:

M20 (N1): My mother, my father and uncle…and my old uncle have been mostly involved [in my recovery]. But it is no good…it is much easier to get in contact with people in the [substance using] community again…if you have a family connection with…or you are the kid of substance users…

These findings are consistent with those of others and our previous findings that participants' most salient challenges were their relationships associated with substance use (Bahl et al., 2019; Pettersen et al., 2018). The main challenge from these relationships was exposure to substances and substance use:

F20 (W7): I fell into a relationship characterised by heavy use of drugs with a guy who was an extreme substance user. First it was just weed, it started there…and it just escalated to pills and acid too… I just had had enough and put my foot down, that this didn't work, that “you are a broken human being who is just ruining me”… So after that it was no more, I did it [used substances] once with some other friends who are still doing it…sniffing, taking pills, smoking and drinking and after that just “no”…

As these quotes illustrate, maintaining relationships with family, partners and friends who themselves use substances can be deeply harmful to the recovery process (Bahl et al., 2019; Pettersen et al., 2018). This issue will be further described in theme four (substance use‐related communities).

#### 5.1.4 Negative influences from significant others affecting recovery

In addition to relationships associated with substance use, family members were described as sources of other negative experiences that adversely influenced the recovery process. Negative experiences included a lack of understanding about substance use, lack of support, trauma, and dysfunctional family relationships. In studies of young people (under the age of 21 years), these factors have also been described as central predictors of substance use and challenges to recovery (Gonzales et al., 2012; Lardier Jr et al., 2017).

I3: Has your family or anyone important [person] in your life been involved, in any way, in the help you have received [to recover]?

F20 (E10): Um. I have kept it kind of hidden. Because…at least my mother, she would be so…I don't know, she gets more stressed than me when I come with problems. So she has kind of not been a kind of help or support that I can, could have utilised. But I do talk to her about it, and I have managed to be rather open when there has been a problem, but I didn't get as much help, kind of, or support either.

I3: Did they [services] assess anything [related to mental health]?

M23 (E6): Yes, anxiety and depression. Because I don't know who my mother is and that has created anxiety and depression for me. And because my dad has been very violent towards me as well.

I3: So, your family has not been involved [in the recovery process]?

M23 (E6): No, not my family.

As can be seen from these excerpts, the same communities that facilitated and supported recovery for some, were for others sources of destructive factors (e.g., lack of parental support and family conflict) that hinder recovery. These findings support a broader, more nuanced view of social recovery capital—including its dark side (Weston et al., 2018). For some, negative social capital can introduce challenges, and even lead to negative recovery outcomes.

### 5.2 Theme 2: Geographical communities

#### 5.2.1 Exposure to substances and substance use

Neighbourhoods and local communities have been identified within the PSOC literature as geographical communities (Perkins & Long, 2002). For specific groups of young people with substance use problems (e.g., Hispanic youth in the USA), neighbourhood is a key moderator of substance use predictors (Lardier Jr et al., 2017). Analysing how participants herein described their geographical communities (e.g., housing and local community) we noticed that they did not report experiencing, or define, their geographical communities as either a PSOC source or a central social system in their recovery. One explanation for this may be that several of these participants still lived with their families, so that their neighbourhood reflects their family's community rather than their own. Another explanation, although not explicitly expressed, could be that these participants held a more neutral PSOC (passive lack of feeling or viewing the community as unimportant) view of their current neighbourhood. Institutions treating substance use problems and municipal housing may also be seen or experienced as temporary geographical communities. One participant staying at a treatment institution (M23 [W6]) described it as a shared community, describing the positive influence of having someone to talk to and create friendships with. However, most participants who were living in these types of communities described these contexts as challenging their recovery, because they meant exposure to substances and substance use:

M20 (N1): When I first was transferred there, I only had a problem with cannabis. And when you are in treatment there is a lot of talk about this and that, right? And you get very curious in the end. You know that substances are not good for you, but people have different angles and perspectives. After I discharged myself, it was kind of like that…I tried different things…

Similar to both our previous findings and others' regarding Norwegian municipal housing for persons with substance use (Bahl et al., 2019; Brekke et al., 2017; Dyb & Holm, 2015), there was a pattern of describing this negative influence (exposure to substances and substance use). As stated by one participant, keeping one's distance from neighbours was a primary way to protect oneself:

I1: Can you tell me a little bit about what kind of contact you have with other people in your everyday life? Family, friends, neighbours?

M23 (W3): I try not to talk too much with the neighbours, as…as I am kind of afraid that they are using, or…I try to keep a distance from them.

During emerging adulthood, moving away from the geographical community in which one grew up is often part of the psychosocial transition to adulthood. For emerging adults with substance use problems, this life phase may entail moving into a temporary geographical community (e.g., municipal housing and treatment institutions). Thus, among this population, geographical PSOC may not be typical for communities like neighbourhoods. Their sense of belonging to such places may also be limited or non‐existent if they are only briefly a residing member. In addition, those who live with family may experience a neutral PSOC or lack of membership in their geographical community.

### 5.3 Theme 3: Ideal communities

The concept of ideal communities was first introduced to PSOC research by Glynn (1981). In our previous study of MPSOC and recovery from substance use problems, ideal communities were identified as meeting places where one could use one's skills and interests to engage with others in meaningful, positive, and creative activities (Bahl et al., 2019). In this study, such experiences could be divided into *actual* communities in which the participants were members of and that were described as ideal for their recovery and as *envisioned* communities in which the participants expressed as ideal communities that they envisioned as needed to recover. Almost all participants herein described actual or envisioned communities, predominantly addressing the former. Meaningful activities with others were a description central to both types of ideal communities.

#### 5.3.1 Meaningful activities with peers

Meaningful activities are vital to ongoing processes of recovery from substance use problems (Nordaunet & Sælør, 2018; Veseth et al., 2021). Several participants described meaningful activities within the services that were ideal for their recovery. These activities were often undertaken with staff or with others also undergoing recovery from substance use problems:

M23 (W3): …there is a music studio there [at the Centre for Mapping and Follow‐up], then there is lunch at eleven thirty every day. It has been good for me, to eat in a social context. And…um… They have yoga there, which has been very good for my body.

I1: What meaning has that had for you?

M23 (W3): I think it has been very meaningful for me. That I have a place to go to every day…I get to know myself better and… It has led me into something called Narcotics Anonymous. That's what I needed.

As this quote illustrates, meaningful activities in actual ideal communities—in this case provided by services—can promote a sense of purpose and forge connections within new communities. Another dominant characteristic of activities that promote participants' recovery processes was that they provided a distraction from substance use:

I4: What kind of help do you consider the most important to you?

M22 (E3): To have something to do. […] Too much leisure time can be harmful.

I4: If you could get—get just the help you want, what would that service look like?

M23 (E9): … Give me a job, or something like a course, that…eh…like, how do I put it? So that I have something to do […] Instead of just standing somewhere with nothing to do, because that is when you start thinking about cannabis, or doing bad things.

As others have identified, meaningful activities were described as introducing positive experiences and joy to the recovery process (Davidson et al., 2006; Veseth et al., 2021).

M18 (E1): I haven't been good at school. I would rather, I think I would have taken a job instead. […] So I think I would have done that instead, and then you kind of forget about cannabis for a bit. If you make it into something fun, you kind of don't have time to smoke cannabis, you know?

Meaningful activities were clearly an important part of participants' recovery processes. However, not only was social capital central to participating in such activities (as illustrated by Theme 1), economic capital also played a central role in the possibility of participating in these communities:

M23 (E9): …I used to play soccer, but eventually it became too expensive. It cost my mother a lot, and she has 10 kids, so she can't pay like 3000 NOK [about 300 USD] for me and then there are nine other kids there. A person living in the west end or a family with two kids, they can afford that kind of thing…

Finally, personal interest was clearly important for defining which activities were experienced as meaningful. However, services did not always satisfactorily promote personally meaningful activities:

M23 (W3): Of course, it is good to work too [for recovery], right? But I think there should be more facilitation for that. For instance, I want to work with music, right? And then I think there should be an effort to ask “How can we help you to work with music?”, right? Be somewhat more active in that, rather than… “You don't have to work with music, kind of, it is too…” Yes, I think they should be better in supporting your visions like…which have been hard to try to achieve.

For the few participants who were satisfied with services' help in staying in contact with others, simply being around peers undergoing recovery seemed to be a central aspect of their understanding of actual‐ideal communities:

I2: Yes, how did you [and the service] work on that [getting in touch with others]?

F20 (W8): No…just being around people going through the same problem…it helps…there is not a lot more to say about that [laughs].

I2: So, just being here with other people who participate in the service is helpful [for you]?

W20 (W8): Yes.

This is consistent with earlier findings suggesting that meaningful activities are important for recovery processes because they promote social recovery capital (e.g., getting in touch with new communities), prevent substance use, and promote new positive experiences (Best et al., 2013; Davidson et al., 2006; Nordaunet & Sælør, 2018; Veseth et al., 2021; Weston et al., 2018). Moreover, these findings support a holistic, “all‐inclusive” approach to meaningful activities in recovery (i.e., including all activities as equal and related to recovery), because personal interests are important for defining what is experienced and understood as meaningful.

While previous reports have highlighted connectedness as critical to the association between participation in meaningful activity and recovery (Nordaunet & Sælør, 2018; Veseth et al., 2021), this did not appear central to the current participants' understanding of recovery. Simply being around peers who share the experience of being in a process of recovery and doing something personally interesting sufficed. This individual‐centred focus may be related to life stage, confirming that emerging adulthood is a period distinguished by preoccupation with oneself and one's needs, during which time there is less interest in social context and community (Arnett, 2012; Gordon et al., 2020; Weiss‐Dagan, et al., 2021). Compared with the general sample in our previous MPSOC and recovery study (Bahl et al., 2019), these emerging adults appear concerned with similarity (i.e., being with peers in recovery) rather than member diversity, which was an important aspect of meaningful activities among the previous sample.

### 5.4 Theme 4: Substance use‐related communities

#### 5.4.1 Exposure to substances and substance use

Substance use‐related communities can be important sources of belonging during the early phase of recovery. However, these relationships are also likely to reinforce substance use (Weston et al., 2018). Herein, we outline the harmfulness of maintaining relationships with others who also have substance use problems. In the initial analysis stages, substance use‐related communities were coded by the first and second authors as a subtheme of relational communities (see Figure 1). The first author also identified substance use‐related communities as part of geographical communities (i.e., treatment institutions, municipal housing). Yet some participants described substance‐using communities (*rusmiljøet*) separately. Given the organising and unified concept within these subtheme descriptions (i.e., family and friends who were part of a substance‐using community, and a substance‐using community per se), these were merged into one theme: substance use‐related communities. These communities were generally described in a negative manner, as frightening and promoting uncomfortable confirmations of participants' substance use problems:

I1: What was it that gave you that motivation [to start the recovery process]?

M22 (W5): …because I was thinking about how much I was getting high and what was happening, and how I got in touch with people in substance‐using communities, and…there was so much going on at that time, or when… So I was almost afraid to keep using substances, kind of. Because I came in contact with people who I normally don't come in contact with. And then….I was afraid of getting high again, so I wanted to…because I started thinking…that I want to do other things than them, kind of, than just getting high and using substances. I found motivation in that.

This participant seems to distance himself from those with whom he came into contact due to his substance use. In fact, this distinctiveness and sense of alienation appeared to motivate initiation of his recovery process. This highlights the association between NPSOC dimensions (i.e., distinctiveness and abstention) and recovery.

#### 5.4.2 Point of departure and NPSOC development

An important peer researcher insight was that substance use‐related communities are the point of departure with respect to recovery processes. These are the communities individuals leave when they begin a recovery process. However, most people with substance use problems are likely to have current or previous positive PSOC experiences within a substance use‐related community (Stevens et al., 2012; Weston et al., 2018). Likewise, several participants described key positive PSOC (i.e., shared experiences and inclusion) aspects of their substance use‐related communities:

I2: What meaning would you say it [the help from services] has had for your situation in life?

F19 (W1): You know, all in all, with substance use and…? I feel that actually…I won't lie. When I was using [substances], I was enjoying myself… You get really good friends and good things happen and things like that, so for that matter, it has been a kind of good experience too…I have never been a hard drug addict, kind of. But…I have done a lot of substances, I have.

At the same time, distancing oneself and abstention from these communities was often central to several of the participants' recovery:

I1: Yes…and you said a little bit about friends and that you have a better group of friends now.

F20 (W7): Yes, I don't have friends who use substances anymore, that…I have distanced myself from that. So, now I just have friends who have movie nights together, just hang together in the city centre and sit at home at each other's place and relax, and that's fine…

I2: Mm…yes…so having a larger and substance‐free community, has that made a big difference [for your recovery]?

F20 (W7): Yes, that has been important.

In addition, establishing new community relationships was central to several of the participants' recovery process:

M23 (E6): Yes, it did [help me], it was very good, something good.

I6: Did you see it at the time, when you where there, or…?

M23 (E6): Not at that moment, and I was kind of occupied with having fun and just being with my new friends, but at the same time I think it was good, because when I was with my “good friends” who were not in a bad environment, I did reasonable things. We danced, ate good food and had fun. I got invited to gaming nights, movie nights, which I would never have been invited to before. So it was kind of a different world, and it was something else than being on the complete other side of the environment.

Another central aspect of this recovery‐related community transition was the participants' motivation for distancing themselves from substances to gain trust and acceptance within their new relationships:

F20 (W7): …they [new friends] are like, if I do it I do it [use substances]. But they don't want to be with me then, I can relate, but I don't want to do it if it makes them not talk to me, and I don't want to lose that trust from my friends.

I2: Yes, so friends are important, that they trust you.

F20 (W7): Mm. Very important.

Consistent with previous evidence (Best et al., 2008; Dingle et al., 2015; Kelly et al., 2014), our findings suggest that maintaining distance and refraining from substance‐related communities—and thereby setting the grounds for an NPSOC to them—was an adaptive, central element in participants building a meaningful life without problematic substance use. Earlier studies have illustrated a close association between the social bonds among recovery community members and social identity, social networks, recovery capital, and quality of life (e.g. Mawson, et al., 2015). Our findings suggest that positive PSOC (e.g., in substance‐using communities) can be both a starting point to moderate and a goal to strive for (e.g., in new recovery‐facilitating communities), to promote and shape a recovery identity, personal resources, and health.

Importantly, social identity and community transition seem vital to a smooth recovery process, in which leaving a substance use‐related community need not lead to a “dead end,” as it did for one participant:

M22 (N3): After I quit using substances, I had to stop seeing a lot of the people I knew. So right now, I sit mostly at home by myself.

Our findings reflect previous research in suggesting that an NPSOC (distinctiveness, abstention, frustration, and alienage) can be destructive and socially isolating as well as adaptive and leading to positive outcomes for those who perceive their substance‐using community as a burden rather than a resource (Brodsky, 1996; Lardier Jr et al., 2020). For emerging adults recovering from substance use, it may be crucial that services support the psychosocial transition from membership in a substance‐using community to one in new, recovery‐facilitating communities, while simultaneously ensuring that this transition does not lead to isolation.

### 5.5 Summary of findings

**Table 2:** Overview of community elements participants described as influencing their recovery processes

Communities	Elements promoting potential for recovery	Elements representing challenges to recovery
1. Relational (family, partners, and friends)	Social resources (e.g., information about services, initiating contact with services, practical and emotional support in contact with services) Motivation for recovery	Exposure to substances and substance use Negative influences (e.g., violence, lack of support, lack of understanding)
2. Geographical (treatment institutions, municipal housing)		Exposure to substances and substance use
3. Ideal (actual and envisioned)	Meaningful activities with peers	
4. Substance use‐related communities	Development of a negative sense of community	Exposure to substances and substance use

*Relational communities* (i.e., family, partners, and friends) promote several important elements for recovery processes. They initiate contact with and supplement services; they also provide practical and emotional support and stimulate motivation for recovery. Yet these relationships can also impede the recovery process, particularly if they involve exposure to substances or substance use, or carry histories of negative experiences such as violence and lack of support or understanding. We did not identify traditional *geographical communities* in participants' descriptions of recovery communities. However, treatment institutions and municipal housing did present challenges through exposure to substances and substance use. *Ideal communities* for recovery were typically described as promoting recovery through meaningful activities with peers. Finally, *substance use‐related communities* (irrespective of being relational or geographical) could, in some cases, promote a specific recovery element: NPSOC development (in particular, alienage, distinctiveness, and abstention). Participants usually described these communities as sources of exposure to substances and substance use, and thus significantly challenged personal recovery.

## 6 LIMITATIONS, STRENGTHS, AND FUTURE RESEARCH

This study adopted a seven‐step reflexive, deductive, and collaborative thematic approach. Three perspectives were included: community psychology, sociology, and personal recovery. Although this triangulation of perspectives enhanced our understanding, it also led to challenges.

First, the authors' perspectives did not impact the analysis process equally. The first authors' community psychological perspective took a dominant role, providing the main theoretical framework. Future studies should consider an autoethnographic approach, to ensure power relation symmetry in future collaborative research (Phillips et al., 2021).

Second, our analytic approach was thorough and inclusive with respect to relevant theoretical perspectives. However, repeated deductive thematic analyses may have caused us to lose the ‘whole story’, reducing some nuances of participants' descriptions and making the findings appear fragmented.

Third, several interviews (13 of 21) were conducted by interviewers who did not participate in the analysis. Although all authors performed individual in‐depth analysis including listening to audio recordings of interviews, adding potentially important interview setting details may have further influenced their understandings. When it is necessary to use multiple interviewers, future studies should also strive to include all interviewers in the analysis process.

Fourth, sample characteristics may have impacted transferability. Aside from the differences between the publicly financed Norwegian welfare system and other Western systems described above, which likely influence how our participants related to their communities, the participants were neither members of education nor employment environments. Thus, most depended on social benefits (although a few reported earning an income by selling illegal substances). Emerging adults with substance use problems who are employed or enroled in formal education may thus have membership and opportunities in different communities from those studied herein. This sample did, however, provide useful information about marginalized emerging adults who are outside school and work communities, fulfilling the call for descriptions of both PSOC and substance use recovery among groups with different resources (Hennessy, 2017; Townley et al., 2011).

Finally, the literature and present findings suggest that future research should more closely examine the associations between positive PSOC, ideal communities, meaningful activities, transitions and trajectories to recovery, and identity development among emerging adults. In particular, associations should be explored between MPSOC (including NPSOC) dimensions and social and negative recovery capital, as well as concepts of ideal communities among different samples with substance use problems. In sum, promoting recovery and preventing substance use problems among emerging adults requires a better understanding of how to leverage both community resources and MPSOC, and how to ameliorate community‐related challenges that emerging adults confront in their personal processes of recovery from substance use.

## 7 CONCLUDING REMARKS

We all depend on social relationships for integration and, consequently, health, and well‐being. We all need to be accepted by, connected with and valued by others throughout our lives (Bahl et al., 2021b; Brewer, 2004; Cicognani et al., 2014; Fiske, 2018; Moore et al., 2018; Sarason, 1974). Thus, it is highly relevant within substance use treatment to learn about individuals' experiences with their social contexts and types of social bonding. However, the effects of social bonding and a sense of community on recovery processes may differ across life phases. The aim of this study was to describe PSOC among emerging adults who are in processes of recovery from substance use problems, and the influences their communities and social environments have on their recovery processes.

To date, the potentials and challenges of different community types for recovery from substance use have been under‐addressed. Thus, we undertook this follow‐up study of emerging adults' experiences with how different types of communities influence their recovery processes. We also examined the evidence pointing to the need for a broader understanding of community belonging for a complete understanding of the ways communities influence recovery, this time among emerging adults (Bahl et al., 2019; Foster & Spencer, 2013; Gordon et al., 2020; Henry & Slater, 2007; Lardier Jr et al., 2017, 2019a, 2019b; Mayberry et al., 2009).

In sum, our findings suggest that relational, geographical, substance use‐related, and ideal communities all influence emerging adults' personal recovery processes in different ways. Our findings confirm prior evidence for the important, supporting, and motivating roles of relational communities (i.e., family, partners, and friends) in recovery, in social recovery capital, and in receiving assistance from services (Gordon et al., 2020; Hogue et al., 2021; Mayberry et al., 2009; Ness et al., 2016; Weston et al., 2018). These data also reflect the central role of ideal communities and meaningful activities with others in recovery from substance use (Bahl et al., 2019; Nordaunet & Sælør, 2018; Veseth et al., 2021). Moreover, the findings reflect earlier results about social resources' potential dark side, indicating that distance and abstention from communities that promote the predictors of substance use (e.g., lack of parental support and family conflict) may be crucial to recovery (Best et al., 2008; Dingle et al., 2015; Kelly et al., 2014; Weston et al., 2018). Future studies of emerging adults may benefit from adopting the broad MPSOC conceptualisation, to gain a more nuanced and complete understanding of community interest and participation than has been possible with the current primarily geographical focus in studies on this distinct developmental stage.

The findings from the present study also contribute new information about belonging and recovery among emerging adults with substance use problems. First, this population's sense of belonging and recovery appear to include NPSOC dimensions such as abstention and alienage, rather than just negative community relationships, as was identified in our previous study with a more general sample (Bahl et al., 2019). Second, geographical communities such as neighbourhoods and local communities, traditionally considered a main PSOC source (Lardier Jr et al., 2017; Perkins & Long, 2002), do not appear central to emerging adults' recovery experiences. Future qualitative studies should use concrete questions about geographical PSOC (for question examples, see Bahl et al., 2021a) to investigate in‐depth whether this reflects this population's neutral PSOC (i.e., a passive lack of feeling) (see Brodsky et al., 2002), the abstention dimension of NPSOC (i.e., uncaring attitude), or as suggested: a life stage‐related lack of interest in geographical communities (Arnett, 2012; Gordon et al., 2020; Weiss‐Dagan et al., 2021). Compared with previous, general samples with substance use problems, this sample reported that simply being around peers who were similar with respect to undergoing a recovery process was a central part of their understanding of meaningful activities, rather than connectedness and community member diversity (see Bahl et al., 2019; Nordaunet & Sælør, 2008; Veseth et al., 2021). Future research should investigate emerging adults' understandings of meaningful activities more deeply, to get a better understanding of necessities for recovery processes during this distinct life period. Finally, our findings indicate that even in the social‐democratic welfare state of Norway, emerging adults' social and economic resources play a central role in which public services are available to them, and thus influence their positive developmental potential. It is thus crucial that community psychologists and professionals working in healthcare services across the world continue to cocreate context‐sensitive research with service users and peer researchers so that we can obtain the broad and deep understanding needed to fulfil our shared agenda of health‐related prevention and promotion.

### 7.1 Practical implications for substance use treatment

Our findings suggest several ways in which substance use treatment approaches (e.g., Asset Based Community Development [Best et al., 2016], network meetings [Seikkula et al., 2018], and MPSOC mapping tools [Bahl et al., 2021a]) might promote social resources and community connections.

First, it is important that these approaches consider age group‐specific aspects of PSOC and recovery. It is essential to acknowledge that emerging adulthood is a period during which individuals with substance use problems may need more motivation and support for community participation and connection, and to join new recovery‐facilitating communities.

Second, a supportive, motivating relational community (i.e., family, partners, and friends) and meaningful activities with peers should be included in a broader, “all‐inclusive” manner. This will likely ensure important social resources are available and facilitate both desired distracting and joyful experiences during treatment. For emerging adults who lack a supportive, motivating relational community, it is important that services assist in psychosocial transitions to new recovery‐facilitating (and preferably peer) communities. Municipal services have a crucial role in this respect, as many emerging adults with substance use problems may never be admitted for specialised treatment.

Furthermore, in using these methods and tools to treat substance use problems, it is crucial that sources of negative experiences (e.g., violence, lack of support, and lack of understanding), NPSOC (distinctiveness, abstention, frustration, and alienage), and positive PSOC in substance use‐related communities are mapped and assessed systematically as part of the initial recovery process. Health services can thus promote several important aspects of service users' recovery, especially various community experiences, including NPSOC. It may also be possible to work systematically on the dark side of PSOC (i.e., exclusion) and prevent isolation or lack of PSOC during between‐community transitions in clinical pathways, particularly between specialised and primary services.

Together, these efforts are likely to promote more involved, coherent, and continuous services and long‐term recovery among a ‘special attention and priority group’ at particular risk of social marginalisation.
